# Avoid the Road to the Poor-House

**Published:** 1903-08

**Authors:** 


### Editorial.

### AVOID THE ROAD TO THE POOR-HOUSE.

There are two closing scenes of life that send an unwelcome shudder to every man: one is the final ending of all humanity, and the other, the exceptional close of life's activity in the place prepared by municipal bounty for the financially wrecked. To the philosopher the first, the inevitable end, is always regarded with the feeling that it is part of the great experiences with which humanity must certainly deal, and, being such, it belongs to and is part of the eternal progress towards increased development, both physically and mentally, here and throughout eternal æons. Death, then, to the wise thinker is the key to unlock the inner sanctuary, the treasure vaults, that hold the undeveloped ideas that become in the centuries the active intellectual life of the coming generations. Hence the typical old man with his scythe is the real benefactor of the race, and, being so, we can leave him to his work of mowing down humanity in order that new men, new measures, new ideas, may become dominant in the world. The man in life is more important than the man in death, and we may leave the latter and deal with the former, to induce him, if possible, to travel away from that path that leads directly to the poor-house, or its equivalent, an old age of want.

Dentistry is not a profession that can amass great wealth. This is only possible with many hands, and the dentist has but two, but properly conducted it will place its votaries into comfortable possessions, and if these be carefully guarded, it must eventually end in a life satisfactory to the individual and those dependent upon his labors.

The thought that dentistry is becoming too crowded is dominating the profession, and in view of the number yearly being graduated from our colleges, the feeling is a natural one and must be accompanied, in the altruistic mind, with the fear that this overcrowding will lead to impoverishment of the many. The writer does not sympathize with this gloomy thought. In a sometime since published article he endeavored to show that the increase in numbers was not in advance of the increase in population, and that all graduates of the present and for many years in the future would be needed.

While this is certainly true, the numbers will force a closer observance of those economics and business methods so helpful in the making of a comfortable life and independent old age.

The impossibility of placing old heads on young shoulders has been demonstrated in all ages of the world, and what is equally difficult is to make young heads believe that vigorous young life will come some day to an end. It is this feeling that the physical activities are a permanent capital that makes the young dentist, as a rule, improvident. His profession furnishes, it may be, all that is needful for comfort, with some of the luxuries, and this fact engenders heedlessness as to that future when energy will have slackened, when the eye will have become dimmed, and the hands will have in great degree lost their mechanical cunning.

The writer has for many years been impressed with the lack of business ability among professional men. It is apparent in all classes, and is not, by any means, confined to dentists. Indeed, as a rule, there are more with business qualifications in this profession than elsewhere, but these will grow fewer as the standard of entrance to dental colleges is increased. The reason for this is not far to seek. The young man graduating from high school or college, and immediately entering a professional school, has had no opportunity for the study of business principles. He passes through the curriculum of his school, graduates, and commences his professional career ignorant of those things upon which success must be attained. Some fail to acquire this knowledge through experience, hence are always loaded with anxious cares, ill luck, as they conceive always has been their portion, and in the end, figuratively, and with some actually, they find the path that leads directly to the poor-house.

It has been said of dentists that when they die their estates consist of a life insurance. It is feared many have not been thus provident.

The experience of a professional life would furnish many illustrations of both sides of this, the provident and improvident. The late Dr. Allport, an excellent example of the first class, for he died comparatively wealthy, mourned, in an article written just prior to his death, this lack of business foresight in dentists. He knew, as we all know, that the man in dentistry who died comfortably well off is not generously distributed throughout the dental profession, but there are sufficient of his quality of mind to cause his example to be of benefit to his co-workers starting out in life. The improvident man, with unbusiness habits, is, unfortunately, plentifully distributed, and his life experience is a disastrous exemplification of the need of something more than a mere professional training to get the best there is out of the world of activity.

The professions are full of sorrowful examples of this latter class. The writer calls up in memory some who have written their names high on the scroll of dental fame, and yet have ended their lives in "Homes," a genteel synonym for the poor-house. Is there anything more depressing than to find one who has lived years and enjoyed the confidence of a large and influential practice at last reduced to penury and dependence on charity. Every one long in practice is familiar with instances of this. The writer has known, alas! too many. The memory of these unfortunates is an ever-present disagreeable reminder of the improvident in dentistry and of the necessity of active work to lessen the growing evil.

The average successful practitioner forgets in the day of success that he is being constantly weighed in the balance by his patients. They grow older with him, and feel that he must be a partaker of the same disabilities from which they suffer. They recognize that age in a dentist is a serious handicap. It may bring wisdom and skill in diagnosis to the medical man, but beyond the sixtieth year there is ever-lessening power to the dentist. The wise parents, feeling thus, will say, in effect, "We must start our children with a young operator, that they may grow up with him," and then the old operator finds his young patients gradually leaving him, and for this he must be prepared.

What, then, is the remedy? Active competition must lead to more thorough teaching, both in theory and practice, and, above all, it must force a different method of procedure on the part of parents and guardians. If the young men after leaving school were placed in a business house or a commercial school for a year they would have a foundation that would make their entire life an easier problem, and then, superadded to this, should be given lectures in our dental colleges on the management of practice, from those who have made the most of their professional lives. It seems to the writer that this has become a most important part of the dental education of the future.

In this connection may the question be asked, Is it not time for the National Dental Association to make some attempt to follow the excellent example of the British Dental Association and establish a fund for the relief, in part, at least, of those who may have been stranded in old age, whether through their own mistakes or through unfortunate conditions? If this body would lead in the formation of a foundation with this object in view, it should be made in a few years available, and under proper precautions would be most valuable in giving the needed help to the most deserving.

A few, doubtless, remember one whose name was a tower of strength in the dental profession. His practice during his days of vigor was of the largest and his clientele the best, but dark days came to him. Unfortunate speculations left him without support in his old age. A few stepped forward under the gracious leadership of one of the true-hearted, and this leader of men was made comfortable for the remainder of his days, not in a poor-house or a home, but with those who could tenderly care for his needs. If this could be accomplished by the few, could not a great association do better? Is the time not ripe for this? If not yet, let not the first quarter of the twentieth century close without some effort being made in this direction. Let it not be said that dentistry was unable to keep a few from joining an ever-increasing procession, marching with haggard mien and faltering limbs on the well-worn pathway that poverty is ever treading towards the homes supplied by municipal and other charities.

